# Ten key considerations for developing interpretable risk prediction models

**DOI:** 10.3389/fbinf.2026.1878681

**Published:** 2026-09-11

**Authors:** Xi Zhang, Wenjie Xu, David R. Smith

**Affiliations:** 1 Department of Biochemistry and Molecular Biology, Dalhousie University, Halifax, NS, Canada; 2 Institute for Comparative Genomics, Dalhousie University, Halifax, NS, Canada; 3 Department of Electrical and Computer Engineering, University of Alberta, Edmonton, AB, Canada; 4 Department of Biology, Western University, London, ON, Canada

**Keywords:** clinical data, explainable artificial intelligence (XAI), fair principles, interpretable machine learning, risk prediction model

## 1 Introduction

### 1.1 Know the enemy and know yourself - Sun Tzu, the art of war

Artificial intelligence (AI) and machine learning (ML) have rapidly transformed clinical risk prediction by enabling high-dimensional data analysis, automated pattern recognition, and individualized risk estimation (Kim et al., 2022). Compared with conventional regression models, ML algorithms can automatically learn nonlinear relationships among diverse clinical variables and optimize predictive performance through hyperparameter tuning (Li et al., 2022). These advances have driven successful clinical applications in diagnostic classification and disease forecasting. Notable examples include a DenseNet-based model for multi-class thyroid disorder prediction using Tc-99m scintigraphy (Ghani et al., 2026), antimicrobial resistance modeling (Ren et al., 2022; Zhang et al., 2025), and the early prediction of infectious threats such as hospital-acquired infections (HAI) (Wang et al., 2024), multidrug-resistant organisms (MDRO) (Zhao et al., 2025), and invasive *Escherichia coli* (Clarke et al., 2024).

Despite these successes, current risk prediction models face persistent limitations. Many models emphasize predictive accuracy while neglecting interpretability, transparency, and reproducibility—factors essential for clinical adoption and ethical deployment (Adnan et al., 2026). To address these methodological gaps, this study proposes a structured workflow for developing Interpretable Risk Prediction Models (IRPMs) that integrate interpretability checkpoints within the established Transparent Reporting of a multivariable prediction model for Individual Prognosis Or Diagnosis (TRIPOD) + artificial intelligence (AI) (Collins et al., 2024) and Prediction model Risk Of Bias ASsessment Tool (PROBAST) + AI (Moons et al., 2025) guidelines. The IRPM framework extends the traditional thirteen-step process for developing, validating, and reporting prediction models by embedding interpretability-specific milestones and facilitating compliance with FAIR principles (Findable, Accessible, Interoperable, and Reusable) (Efthimiou et al., 2024). The methodological foundation of this workflow is illustrated through a recent case study on early prediction of *E*. *coli* infection among Intensive Care Unit (ICU) patients, conducted using the large-scale Medical Information Mart for Intensive Care IV (MIMIC-IV) dataset (Yang et al., 2025).

In this study, Yang et al. (2025) applied Least Absolute Shrinkage and Selection Operator (LASSO) + Boruta for feature selection and built eight ML models, identifying the support vector machine (SVM) as the most optimal model (Area Under Receiver Operating Characteristic Curve (AUC) = 0.745; 95% Confidence Interval (CI): 0.726–0.764). It should be noted that although the SVM achieved the best discrimination among evaluated models (AUC = 0.745), this level of performance remains modest for clinical deployment and highlights the importance of complementary evaluation using calibration, clinical utility analyses, and external validation. Model interpretability was evaluated with Shapley Additive Explanations (SHAP), revealing gender, age, sepsis, sedative use, and potassium levels as key predictors. The team subsequently deployed a web-based *E. coli* Predictor tool to enable real time risk estimation, demonstrating the practical utility of interpretable AI in critical care settings. By aligning interpretability principles with standardized reporting frameworks and integrating lessons from this case study, IRPMs can advance responsible and trustworthy AI in healthcare. It should be noted that the study is presented as an illustrative example rather than as the basis for the framework itself. The IRPM recommendations are intended to generalize across clinical prediction settings. For example, prior work (Piccininni et al., 2024) has shown that different class-imbalance correction strategies can substantially alter calibration and discrimination performance, highlighting the importance of reporting resampling decisions transparently.

## 2 Discussion

### 2.1 Know the novelty of the study and whether it outperforms existing approaches

Recent international guidelines such as TRIPOD + AI (Collins et al., 2024) and PROBAST + AI (Moons et al., 2025) set comprehensive standards for reporting and evaluating AI-driven prediction models. They focus primarily on methodological completeness and risk-of-bias assessment rather than on interpretability workflows. The IRPMs workflow complements these by introducing three operational interpretability safeguards: (i) an interpretability checkpoint, (ii) explanation stability testing, and (iii) causal plausibility assessment (Table 1; Supplementary Table S1). The interpretability checkpoint requires investigators to verify that explanation methods generate clinically coherent feature attributions and provide both global and local explanations for review; models should be reconsidered when explanations are not reproducible or are dominated by clinically implausible features without justification. Explanation stability testing evaluates whether explanation outputs remain consistent across repeated analyses, for example, by repeating SHAP calculations over 1,000 bootstrap samples or multiple random seeds; models fail when highly influential features exhibit substantial rank instability or inconsistent effect directions. Causal plausibility assessment examines whether influential predictors identified by explainability methods are consistent with established biological or clinical knowledge, ideally supported by directed acyclic graphs (DAGs) or structural causal models; models fail when key explanations depend predominantly on predictors lacking plausible mechanistic justification.

**TABLE 1:** Summary of the IRPMs study: ten key considerations for developing interpretable risk prediction models.

No.	Key point to check	Guideline alignment with TRIPOD + AI (Collins et al., 2024) and PROBAST + AI (Moons et al., 2025)	Recommended tools or methods	Example application (Yang et al., 2025)
1	Know the novelty and contribution	Articulate contribution and complementarity with existing standards (TRIPOD + AI items 3a–4; PROBAST + AI risk-of-bias domains)	Comparative literature review; TRIPOD + AI checklist	IRPM introduces an interpretability checkpoint, explanation stability testing, and causal plausibility assessment as additional safeguards
2	Understand data and prevent data leakage	Ensure transparent data handling and partitioning (TRIPOD + AI items 5–12)	Early train/validation split; independent imputation (missForest); correlation filtering (Spearman, Cramér’s V); data dictionary creation	Yang et al. used MIMIC-IV data, missForest imputation, and a 70/30 split; leakage avoided via within-fold resampling
3	Handle class imbalance	Maintain fairness and calibration (TRIPOD + AI item 13; item 14 fairness)	Undersampling/SMOTE/Hybrid methods; cost-sensitive learning; prevalence-aware threshold optimization; multi-center or federated data collection	Undersampling of majority class (7.9% positive) improved calibration and DCA; future multi-center data recommended
4	Select appropriate models	Choose model type balancing interpretability and performance (TRIPOD + AI item 12c)	LASSO/Elastic net/Ridge regression; tree-based ensembles (RF, XGBoost); Kernel methods (SVM); SHAP/LIME for explanation	LASSO + Boruta for feature selection; chose the model achieved best discrimination
5	Tune hyperparameters transparently	Promote reproducibility and transparency (TRIPOD + AI items 12c and 18e–18f)	Grid search/Randomized search/Bayesian optimization; cross-validation (5 folds); publish tuning scripts and parameter ranges	Grid search with cross-validation; optimal SVM kernel and regularization documented
6	Evaluate the model statistically and clinically	Evaluate discrimination, calibration, and clinical utility (TRIPOD + AI item 12e and 23a)	AUC + AUPRC with bootstrapped CIs; calibration curves; decision curve analysis (DCA); clinical impact curves (CIC); subgroup fairness analysis	SVM model: AUC, AUPRC; DCA and CIC confirmed positive net benefit; calibration slope ≈1, intercept ≈0
7	Interpret features via XAI tools	Achieve interpretability and fairness (TRIPOD + AI items 12e, 14, 25)	SHAP (global + local); LIME; Permutation importance; Partial Dependence plots (PDPs); counterfactual explanations	SHAP identified gender, age, sepsis, sedative use, potassium as top predictors; visualized via Shiny-based web app
8	Apply the FAIR principles	Ensure open science and reproducibility (TRIPOD + AI items 18a–18f; Adherence tool scoring)	Public GitHub/Zenodo repository; metadata + DOI assignment; License declaration (MIT, CC BY 4.0); reproducibility checklist (DOI: https://doi.org/10.5281/zenodo.21420568)	Publishing SVM–SHAP code and MIMIC-IV variable metadata for FAIR compliance
9	State limitations clearly	Transparently report bias and trade-offs (TRIPOD + AI item 26; PROBAST + AI quality domains)	Bias assessment; missing-data impact analysis; overfitting risk estimation; interpretability-accuracy trade-off discussion	Single-center MIMIC-IV dataset limits generalizability; undersampling may cause information loss
10	Plan future directions	Outline ethical AI and regulatory readiness (TRIPOD + AI items 27a–27c)	Interpretable deep learning (ANNs, CNNs, RNNs); attention mechanisms, concept activation vectors; federated learning for multi-center data; ethical frameworks (e.g., JustEFAB, FDA, health Canada)	Future IRPMs should integrate multimodal EHR + microbiology data and fairness auditing for real-world deployment

**FIGURE 1:**
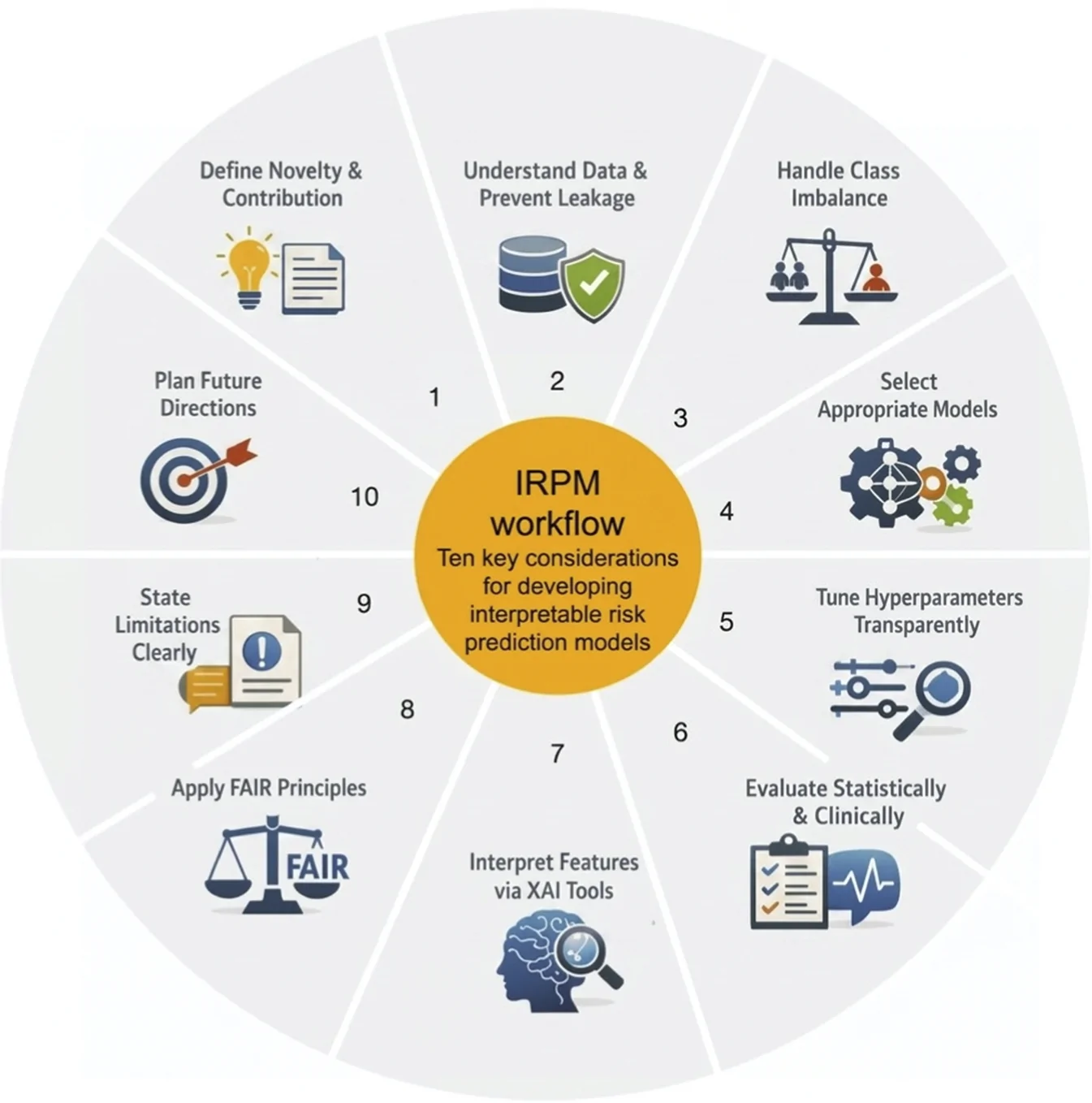
Ten things to consider in conceptual interpretable risk prediction models (IRPMs) development workflow.

Applied to the study of Yang (Yang et al., 2025), IRPMs clarify the novelty of how their ML model + SHAP pipeline aligns with TRIPOD + AI Items 12e (performance + interpretability) and 18e–18f (open science) (Collins et al., 2024), while highlighting areas for improvement, such as subgroup fairness analysis and external validation.

Specifically, Yang and colleagues first assessed the current risk of *E*. *coli* infection in ICU patients, and then reviewed prior studies on early prediction using cephalosporins and invasive procedures (Yang et al., 2025). They identified an urgent need for robust, clinically interpretable ML models specifically tailored to ICU populations. When designing a study, it is crucial to clearly detail the novelty: whether your model outperforms existing approaches or demonstrates innovation in interpreting clinical variables for easier infection surveillance. Employing mature, validated ML algorithms (e.g., logistic regression, random forest, and other classification models) is encouraged to enhance transparency and clinical credibility. Overall, when designing a study, researchers should articulate novelty explicitly—whether the model introduces an innovative interpretability approach or enhances clinical usability. The IRPMs workflow operationalizes these principles by translating TRIPOD + AI’s high-level reporting recommendations into actionable modeling steps.

### 2.2 Know your data and how to prevent data leakage

Data leakage is when information from validation or test sets inadvertently influences model training, which remains one of the most common pitfalls in clinical ML. Preventing data leakage is essential for maintaining model validity. A common practice is to use temporal validation, establishing a strict chronological split between your derivation and validation datasets (Ramspek et al., 2021). The guidelines of TRIPOD + AI at Items 5–12 mandate transparent reporting of data sources, preprocessing, and partitioning (Collins et al., 2024).

In their *E. coli* study (Yang et al., 2025), Yang et al. extracted demographic, laboratory, comorbidity, and treatment data for 52,554 ICU patients from MIMIC-IV. Missing values were imputed using the missForest algorithm (Stekhoven and Bühlmann, 2012), and undersampling for class balancing (Piccininni et al., 2024) was performed strictly within training folds during cross-validation to ensure independence. Best practices for preventing leakage include the following: Researchers should split the datasets (e.g., 70% training, 30% validation) prior to preprocessing and then impute missing values separately within each subset, which can maintain complete independence between the training and validation data. Lastly, the clinical feature selection and class balancing steps should be executed within the training data. Additionally, Yang et al.’s correlation filtering (Spearman >0.6, Cramér’s V > 0.5) and the intersection of 28 variables improved interpretability and reduced multicollinearity, consistent with TRIPOD + AI at Item 12a (“Describe how the data were used in the analysis”) (Collins et al., 2024).

### 2.3 Know what to do with class imbalance and try to collect more data sources

Class imbalance is where positive outcomes are much fewer than negative ones, which can distort performance and fairness. TRIPOD + AI (Item 13) requires justification for resampling and recalibration (Collins et al., 2024). Ideally, the clinical data can be collected from different hospitals or countries, but due to the privacy of the patients and policy of different countries, it could be difficult to realize multi-center validation, which might weaken the model’s applicability on a broad scale. In Yang et al.’s dataset (Yang et al., 2025), *E. coli* infection represented 7.9% of cases (4, 157 patients). The authors applied undersampling to balance classes while preserving data authenticity. Alternative approaches include Synthetic Minority Over-sampling Technique (SMOTE), Oversample using Adaptive Synthetic (ADASYN), cost-sensitive learning, and prevalence-aware threshold optimization. While oversampling can improve sensitivity, it risks introducing synthetic artifacts. Undersampling maintained real-world structure and yielded strong calibration and Decision Curve Analysis (DCA) results. Future IRPMs studies should pursue multi-center data collection or federated learning to mitigate imbalance and enhance generalizability, aligning with TRIPOD + AI (Item 16) (“differences between development and evaluation data”) (Collins et al., 2024).

### 2.4 Know which models can be fitted in your clinical data

A common question for researchers performing machine learning is whether regression or classification models should be chosen. Model selection must balance interpretability and predictive performance. TRIPOD + AI (Item 12c) (Collins et al., 2024) requires justification and documentation of modeling rationale. Yang et al. used LASSO regression for feature selection and developed eight algorithms including logistic regression (LR), K-nearest neighbors (KNN), decision tree (DT), random forest (RF), extreme gradient boosting (XGBoost), light gradient boosting machine (LightGBM), support vector machine (SVM), and neural network (NNet) (Yang et al., 2025). LASSO improved interpretability during feature-selection by identifying a reduced subset of predictors; however, the final deployed classifier was an SVM, whose interpretation required post-hoc explanation methods such as SHAP. Future IRPMs should report model type, tuning strategy, hyperparameter ranges, validation variance, and rationale for algorithmic choices.

Specifically, for regression, there are logistic regression, Ridge regression, and LASSO regression. In Yang’s work, LASSO regression was chosen instead of standard logistic or Ridge regression, because it integrates both variable selection and regularization within a single framework through an L1 penalty. While logistic regression assumes that all predictors are relevant and includes them in the model, it can lead to overfitting and reduced interpretability when the number of predictors is large or when multicollinearity exists. Ridge regression applies an L2 penalty and keeps all variables. Overall, LASSO can automatically shrink less informative coefficients to zero, producing a sparser and more interpretable model that retains only the most predictive features. Thus, LASSO offers effective feature selection capability and model simplicity, which are attributes particularly desirable in high-dimensional clinical data modeling (Laufer et al., 2023; Tibshirani, 1996).

### 2.5 Know the hyperparameter tuning strategies

To ensure reproducibility through transparent hyperparameter tuning, some researchers prefer to use nested cross-validation (nCV) (Parvandeh et al., 2020). In this approach, the classification model and features for each outer fold are selected based on which combination achieves the highest AUPRC or balanced accuracy during inner-fold validation. TRIPOD + AI (Item 12c) mandates reporting of all model-building steps and tuning procedures (Collins et al., 2024). Yang et al. used grid search and k-fold cross-validation, emphasizing reproducibility over exhaustive optimization (Yang et al., 2025). Alternative strategies include randomized search, Bayesian optimization, and ensemble stacking, which can improve performance but introduce stochasticity. The TRIPOD + AI Adherence Tool (Collins et al., 2024) provides scoring rules for reproducibility, encouraging researchers to publish tuning scripts, fixed random seeds, and parameter grids. Future IRPMs should include hyperparameter tables, validation variance, and rationale for algorithmic choices to enable replication.

### 2.6 Know the appropriate statistical analysis of the model

Area Under the Curve (AUC) and the Receiver Operating Characteristic curve (ROC) are commonly used to evaluate the model’s performance with a 95% Confidence Interval (CI). TRIPOD + AI (Item 12e) recommends reporting discrimination, calibration, and clinical utility (Collins et al., 2024). Other performance metrics, including sensitivity, specificity, F1-score, accuracy, and balanced accuracy, should also be reported. Their uncertainty can be estimated using bootstrap resampling (e.g., 1,000 resamples). It should be noted that due to class imbalance in clinical data, metrics such as Area Under the Precision-Recall Curve (AUPRC) or Average Precision are often more informative than AUC.

Besides, the model’s performance can be impacted by some uncertainties, for research using early-stage clinical data to predict *E. coli* infection during admission (e.g., duration of antibiotics and catheter use) (Yang et al., 2025), the limited availability of variables and the required timeliness of the prediction significantly increase the difficulty of improving the model’s performance. Therefore, when the AUC of the model is not exceptionally high, it is still worth examining if the model’s utility is further supported by its strong performance on other critical metrics, including a good calibration curve and a positive net benefit in the Decision Curve Analysis (DCA), which are vital for clinical applicability. In summary, Yang et al. assessed AUC, AUPRC, F1 score, balanced accuracy, calibration curves, DCA, and Clinical Impact Curves (CIC). For IRPMs, recommended analyses include AUC + AUPRC with bootstrapped 95% CIs, calibration plots (slope ≈1, intercept ≈0). But predictive accuracy alone does not ensure clinical validity. Future IRPMs should integrate causal modeling frameworks (e.g., Structural Causal Models and directed acyclic graphs (DAGs)) to verify mechanistic plausibility.

### 2.7 Know how to extract clinical features using explainable AI (XAI)

Interpretability can transform statistical predictions into clinical insights. TRIPOD + AI (Items 12e and 14) require transparent explanation and fairness evaluation (Collins et al., 2024). When the best model has been chosen with robust calibration and clinical utility supported by decision analyses, the clinical features can be interpreted via SHAP (SHapley Additive exPlanations) (Mosca et al., 2022) or Local Interpretable Model-Agnostic Explanations (LIME) (Zafar and Khan, 2021) and others. For example, Yang et al. (Yang et al., 2025) identified 28 clinically relevant features from 52,554 patients with *E. coli* infections in the ICU. The model interpretability was achieved through Shapley Additive Explanations (SHAP), which decomposed predictions into additive feature contributions, revealing gender, age, sepsis, sedative use, and potassium levels as the most influential predictors. SHAP can provide both global interpretability (population-level feature importance) and local interpretability (individual-level reasoning). Interpretability can be categorized as intrinsic (inherent transparency, as in logistic regression or decision trees) and post-hoc (external explanation of complex models such as ensembles or neural networks) (Supplementary Table S2).

To strengthen interpretability and confirm robustness, SHAP can be complemented with other XAI methods (Supplementary Table S3), such as LIME which builds local linear approximations for individual predictions; Permutation Importance which quantifies how randomizing a feature affects model accuracy; Partial Dependence Plots (PDPs) which visualize marginal effects of features on outcomes; DeepLIFT (Deep Learning Important FeaTures) which highlights neuron-level contributions in deep architectures. SHAP can help identify which variables contribute most strongly to model predictions and can provide both global and patient-level explanations of model behaviour. However, these explanations should be interpreted cautiously. Importantly, SHAP explanations quantify how features contribute to model predictions and should not be interpreted as evidence of causal effects. Features identified as important by SHAP may reflect associations, confounding, or data artefacts. Assessment of causal mechanisms therefore requires separate causal inference approaches, such as DAGs or structural causal models (SCMs), and is not a property of SHAP itself.

A continuing debate in clinical AI concerns whether post-hoc explanations are sufficient for high-stakes decision-making or whether inherently interpretable models should be preferred. Interpretable models such as logistic regression and decision trees provide transparency by design but may sacrifice predictive performance in some settings. Conversely, black-box models coupled with SHAP or LIME can achieve strong predictive accuracy while providing explanations, although explanation instability and approximation error remain recognized limitations. In the context of IRPM, post-hoc explainability is considered acceptable when accompanied by rigorous stability testing, transparent reporting, and clinical validation; however, inherently interpretable models should remain an important benchmark when predictive performance is comparable.

Validation of explanations is also critical. For explanation stability testing, investigators may repeat SHAP analyses across 1,000 bootstrap resamples or multiple random seeds and compare feature rankings, flagging models when top-ranked predictors exhibit substantial instability. An interpretability checkpoint should additionally verify that both global and local explanations are available and clinically coherent. Finally, causal plausibility assessment should examine whether influential predictors identified through XAI methods are consistent with established biological or clinical knowledge, ideally using DAGs or structural causal models.

Finally, Yang et al. integrated SHAP visualizations into a Shiny web tool (https://predicti.shinyapps.io/ecoli_predictor/) for individualized risk estimation, exemplifying how interpretability can support real-time clinical decision-making and open-science reproducibility. The future IRPMs should employ a multi-method, stability-tested, and clinically contextualized approach to interpretability.

### 2.8 Know how to apply the FAIR principle on source codes

It is important to apply the Findable, Accessible, Interoperable, and Reusable (FAIR) principles for bioinformatics and open-source tools. TRIPOD + AI (Items 18a–18f) emphasize the FAIR principles (Collins et al., 2024). FAIR in bioinformatics means making data, tools, and training findable, accessible, interoperable, and reusable, following principles that boost data sharing, discovery, and integration for machines and humans. If online IRPMs do not include the source code or parameters, it will limit the study’s reproducibility and transparency. The common approach is to make the code publicly available in a permanent online GitHub repository, detail the web tool with a help documentation page, providing detailed instructions for variable input, model use, and result interpretation, as well as a feedback page to allow users to report issues or suggestions for future optimization. Yang et al. could strengthen FAIR compliance by publishing their SVM–SHAP pipeline and MIMIC-IV metadata on GitHub (Yang et al., 2025). FAIR implementation ensures reproducibility and long-term accessibility. A detailed reproducibility checklist (DOI: https://doi.org/10.5281/zenodo.21420568) covering study design, data processing, model development, performance evaluation, and FAIR compliance is provided in Supplementary Table S4.

### 2.9 Know the limitations

Every IRPMs must transparently acknowledge constraints. It is important to mention the limitations and compare the relevant work to understand the stage at which your study currently stands and how it may be improved in the future. TRIPOD + AI (Item 26) stresses that transparent discussion of limitations contextualizes results and strengthens credibility. For example, in the study of Yang et al., the *E. coli* infection patients were derived from a single-center database (MIMIC-IV), which lacks external, multi-center validation, thereby limiting the generalizability to other healthcare systems with different patient characteristics and microbiological epidemiology. No method is perfect. Common limitations include bias arising from missing data and imputation assumptions, the risk of overfitting despite cross-validation, trade-offs between interpretability and predictive performance, lack of external validation, and the inability to establish causal relationships.

### 2.10 Know the future perspective

Due to the workload or data limitations, it is not always possible to apply fully robust, validated methods to develop a robust, interpretable, and clinically applicable tool. As datasets continue to grow, deep learning approaches, such as artificial neural networks (ANNs), convolutional neural networks (CNNs), and recurrent neural networks (RNNs), could be leveraged to automatically learn high-order nonlinear relationships among complex clinical and microbiological variables. Importantly, recent advances in interpretable deep learning, including attention mechanisms, concept activation vectors, and counterfactual explanation frameworks, have improved the interpretability of deep learning models without necessarily compromising predictive performance. Future IRPMs should aim to combine predictive power with explanation stability and causal plausibility, rather than viewing complexity and interpretability as opposing forces. Future IRPMs will likely evolve toward multimodal foundation models integrating structured electronic health record (EHR) data, microbiology reports, imaging, and genomics through self-supervised representation learning. Federated training frameworks may further enhance generalizability across institutions while preserving patient privacy. Future IRPMs should include ethical frameworks (e.g., JustEFAB (Mccradden et al., 2023), US Food and Drug Administration (FDA) and Health Canada guidance) for deployment. Importantly, future systems should aim to prioritize causal robustness, fairness auditing, and prospective validation to ensure real-world clinical reliability. Most importantly, future IRPMs should combine predictive accuracy with explanation stability, fairness, and clinical usability, fully aligned with TRIPOD + AI’s open-science ethos.

## 3 Conclusion

The IRPMs study bridges methodological rigor and interpretability within the evolving standards of TRIPOD + AI and PROBAST + AI. While TRIPOD + AI defines what to report and PROBAST + AI defines how to assess quality, IRPMs provide practical guidance for building interpretable models responsibly. Applied to the *E. coli* case study, IRPMs highlight methodological excellence and opportunities for improvement, including subgroup fairness, external validation, and FAIR-compliant code release. By embedding interpretability into model design and reporting, IRPMs aim to promote transparent, equitable, and clinically trustworthy AI systems. Together, these framework and workflow support a shared goal: interpretable, reproducible, and ethically deployable prediction models that enhance clinical decision-making and patient safety.
