# Supplementary material for: Ten key considerations for developing interpretable risk prediction models
Source: Front Bioinform. 2026 Sep 11;6:1878681. doi: 10.3389/fbinf.2026.1878681 (PMC13612535; doi:10.3389/fbinf.2026.1878681)
Supplement: Supplementary file 1 [file Table1.docx]

Ten Key Considerations for Developing Interpretable Risk Prediction Models

Xi Zhang^1,2*^, Wenjie Xu^3^, David R. Smith^4^

^1^Department of Biochemistry and Molecular Biology, Dalhousie University, Halifax,

Nova Scotia, B3H 4R2, Canada.

^2^Institute for Comparative Genomics, Dalhousie University, Halifax, Nova Scotia,

B3H 4R2, Canada.

^3^Department of Electrical and computer engineering, University of Alberta, Edmonton, T6G 2R3, AB, Canada

^4^Department of Biology, Western University, London, Ontario, N6A 5B7, Canada.

*Correspondence [Xi.Zhang@dal.ca](mailto:Xi.Zhang@dal.ca) (X. Zhang)

Keywords: Interpretable machine learning, risk prediction model, FAIR principles, clinical data, Explainable Artificial Intelligence (XAI)

**Supplementary Tables**

**Table S1 | Comparison of different framework or workflow.**

| Framework | Core Focus | Interpretability Coverage | FAIR and Reproducibility Emphasis |
| --- | --- | --- | --- |
| TRIPOD + AI [10] | Transparent reporting of regression or ML‑based prediction models; fairness and open science. | Moderate: Items 3c, 12e, 14 mention interpretability and fairness but lack implementation guidance. | Strong: requires protocol registration, data/code sharing, and open access (Items 18a–18f). |
| PROBAST + AI [11] | Risk‑of‑bias and applicability assessment for prediction models using regression or AI methods. | Moderate: focuses on bias detection, not explanation. | Moderate: fairness and transparency in evaluation. |
| IRPMs (this work) | Methodological framework for interpretable, reproducible clinical prediction models. | High: defines global vs local, integrates SHAP, LIME, PDP. | Strong: embeds FAIR principles and reproducibility documentation (checklist). |

**Table S2 | Comparison of interpretability dimensions in interpretable risk prediction models (IRPMs).**

| Interpretability Dimension | Definition / Scope | Underlying Mechanism or Approach | Advantages and Clinical Utility | Limitations and Validation Considerations | Illustrative Example [13] |
| --- | --- | --- | --- | --- | --- |
| Global Interpretability | Explains overall model behavior across the entire population; identifies which predictors most influence predictions overall. | Aggregate feature‑importance analysis using SHAP summary plots, permutation importance, or PDPs. | Enables population‑level risk factor identification and policy‑level insights; supports fairness audits and variable selection. | May overlook patient‑specific variability; sensitive to multicollinearity among predictors; requires stability testing. | Global SHAP analysis revealed gender, age, sepsis, sedative use, and potassium level as dominant predictors of *E. coli* infection. |
| Local Interpretability | Explains the reasoning behind a single prediction for an individual case. | Case‑specific visualizations such as SHAP force plots, waterfall plots, or LIME local surrogates. | Provides individualized risk explanation; supports clinician–patient communication and bedside decisions. | May be unstable under sampling noise or highly nonlinear interactions; limited generalization. | Local SHAP plots showed how specific patient features (e.g., age = 80, maleative use) shifted infection risk. |
| Intrinsic Interpretability | Transparency is inherent to model structure (e.g., logistic regression, decision trees). | Coefficients, rules, and feature weights directly interpret relationships; no additional explanation tool required. | Simple, transparent, and easy to validate; low computational cost. | Limited capacity to capture complex nonlinear patterns; potential reduction in predictive accuracy. | Logistic regression coefficients and decision‑tree rules clarified associations between sepsis and infection risk. |
| Post‑hoc Interpretability | External techniques applied after training to explain black‑box models (ensemble, kernel, or neural networks). | SHAP, LIME, PDPs, Permutation Importance, and DeepLIFT (for deep architecture). | Enables interpretation of complex models with high predictive power; facilitates fairness and causal plausibility analysis. | Approximation errors; computational cost; requires explanation stability validation and causal plausibility checks. | SHAP and PDP visualizations were used to interpret the SVM model in *E. coli Predictor*, translating predictions into clinical logic. |

**Table S3 | Comparison of explainable AI (XAI) techniques in interpretable risk prediction models (IRPMs).**

| Method | Interpretability Type | Core Principle / Strengths | Limitations | Typical Clinical Use Case |
| --- | --- | --- | --- | --- |
| SHAP (Shapley Additive Explanations) | Global + Local, Post‑hoc | Based on cooperative game theory; provides additive, consistent attributions; applicable to any model type. | Computationally intensive; unstable with highly correlated predictors; requires stability validation. | Used in *Yang et al.* [13] for both global and individual‑level explanation; supports visualization in the *E. coli Predictor* web tool. |
| LIME (Local Interpretable Model‑Agnostic Explanations) | Local, Post‑hoc | Builds simple linear surrogate models around a single prediction; intuitive for clinicians. | Sensitive to sampling noise; less reliable for nonlinear interactions; lacks global perspective. | Case‑based interpretability to explain outlier or critical patient predictions. |
| Permutation Importance | Global, Post‑hoc | Measures change in model accuracy after random shuffling of each feature; straightforward and model‑agnostic. | Overestimates correlated features; does not indicate effect direction. | Cross‑check feature relevance to confirm SHAP rankings. |
| Partial Dependence Plots (PDPs) | Global, Post‑hoc | Displays marginal effect of a feature (or pair) on predicted probability; useful for monotonic trends. | Assumes feature independence; misleading when strong interactions exist. | Visualizes clinical trends (e.g., higher potassium → greater infection risk). |
| DeepLIFT (Deep Learning Important Features) | Local + Global, Post‑hoc | Decomposes neural‑network output by comparing activations to a reference baseline; identifies neuron‑level contributions. | Applicable primarily to neural networks; requires defined reference states; less suited for tree‑based models. | Valuable when extending IRPMs to deep‑learning architectures for multimodal data (e.g., EHR + imaging). |

**Table S4 | Reproducibility checklist for interpretable risk prediction models (IRPMs).**

| **Category** | **Checklist Item** | **Purpose and Action** | **Y/N** |
| --- | --- | --- | --- |
| **Study Design and Protocol** | 1. Provide study title, objectives, and research question. | Clarifies scope and intended outcome. |  |
|  | 2. Register protocol and metadata dataset DOI (e.g., OSF, Zenodo). | Ensures findability and traceability of study materials. |  |
| **Data Description and Access** | 3. Specify data source (e.g., MIMIC‑IV or institutional dataset) and access link. | Allows data verification and reuse. |  |
|  | 4. Describe data partitioning (train/validation/test) and sample size. | Prevents data leakage and bias. |  |
|  | 5. List all variables used, definitions, units, and missing‑data handling method. | Supports data reconstruction and consistency. |  |
| **Feature Selection and Preprocessing** | 6. Provide code for feature selection methods (e.g., LASSO, Boruta, SVM‑RFE). | Reproduces variable selection steps. |  |
|  | 7. Report software versions and libraries used (e.g., R 4.4.2, Python 3.9, scikit‑learn 1.6.1). | Ensures computational compatibility. |  |
| **Model Development** | 8. Specify model types and hyperparameters for each algorithm. | Enables exact replication of model training. |  |
|  | 9. Provide random seeds and cross‑validation details (k‑fold number, stratification rules). | Guarantees consistent results across runs. |  |
| **Performance Evaluation** | 10. Publish scripts for calculating AUC, AUPRC, F1, calibration, DCA, and CIC. | Validates reported performance metrics. |  |
|  | 11. Include bootstrapping or cross‑validation statistics (≥ 1 000 resamples). | Quantifies statistical uncertainty. |  |
| **Interpretability and Fairness** | 12. Share code for SHAP, LIME, or PDP visualizations and subgroup fairness analysis. | Reproduces interpretability outputs. |  |
|  | 13. Provide example plots, saved models (.rds) or JSON outputs for global and local explanations. | Demonstrates model transparency. |  |
| **Open‑Science and FAIR Compliance** | 14. Deposit data and code in public repositories (GitHub/Zenodo) with permanent DOIs. | Facilitates reuse and peer verification. |  |
|  | 15. Include license information (MIT or CC BY 4.0) and contact for user support. | Clarifies reuse rights and responsibility. |  |
| **Limitations and Ethical Considerations** | 16. Discuss bias, missing data, and overfitting risks and how they were addressed. | Ensures ethical and transparent reporting. |  |
|  | 17. Provide link to institutional ethics approval and data‑use statement. | Confirms compliance with ethical standards. |  |
| **Future Usability** | 18. Document how the model can be re‑executed or integrated into clinical workflow (web app or API). | Supports translation to practice. |  |
